# Effects of a Workplace Sit–Stand Desk Intervention on Health and Productivity

**DOI:** 10.3390/ijerph182111604

**Published:** 2021-11-04

**Authors:** Jiameng Ma, Dongmei Ma, Zhi Li, Hyunshik Kim

**Affiliations:** 1 Faculty of Physical Education, Sendai University, Shibata 9891693, Japan; jm-ma@sendai-u.ac.jp (J.M.); dn-ma@sendai-u.ac.jp (D.M.); s21510211@sendai-u.ac.jp (Z.L.); 2 Department of Medicine and Science in Sports and Exercise, Graduate School of Medicine, Tohoku University, Sendai 9808576, Japan

**Keywords:** sit–stand desk, sedentary behavior, workplace intervention, work productivity, behavior change

## Abstract

In Japan, standing while working has not yet become commonplace, and there is little evidence to support the benefits of standing during the workday. Therefore, this study assessed the relationship between the introduction of a sit–stand desk and its ability to reduce the negative effects of sitting too long and increase employees’ general health and productivity. Seventy-four Japanese desk workers participated in this three-month intervention study. Using a randomized controlled trial, the participants were divided into intervention (*n* = 36) and control (*n* = 38) groups. The participant characteristics were ascertained using a questionnaire. The intervention effectiveness was assessed by measuring health-, physical activity-, and work-related outcomes. The results indicate that the intervention group significantly decreased their sitting time at work (*p* = 0.002) and had reduced neck and shoulder pain (*p* = 0.001). There was a significant increase in subjective health (*p* = 0.002), vitality in work-related engagement (*p* < 0.001), and self-rated work performance over a four-week period (*p* = 0.017). These findings indicate a significant difference between the two groups, demonstrating the effectiveness of a sit–stand desk in reducing sedentary behavior and improving workers’ health and productivity. Future research can accumulate further evidence of best practice use of sit–stand desks.

## 1. Introduction

Many studies have demonstrated that prolonged sedentary behavior (SB) increases health risks. For example, a systematic review associating SB and health outcomes found that prolonged SB in adults was associated with a 1.18-fold increased risk of cardiac death, 1.17-fold increased risk of cancer death, 1.91-fold increased risk of diabetes, and 1.24-fold increased risk of all-cause death [1]. Additionally, in a 12-year follow-up study examining the relationship between sitting time and the risk of death from cancer in women aged 50 years and older, the risk of death from cancer was 1.21 times increased for those who sat for eight hours per day, compared to those who sat for less than four hours per day [2]. Despite this evidence, it has been reported that SB accounts for approximately 60% of daily waking hours, while moderate- to vigorous-intensity physical activity (MVPA) accounts for approximately only 5% of adults’ waking hours [3].

According to a survey on SB conducted across 20 countries worldwide, Japanese people sit for the longest time, averaging seven hours a day [4]. As Japanese workers have long working hours, the sitting time of office workers is believed to be similar; the effect of which could be significant to their health. Consequently, the Ministry of Health, Labor and Welfare of Japan has identified the current physical and mental health of workers as a problem, leading them to define measures for “health management” and “work reform”. Recently, to improve productivity by enabling workers to work energetically in the workplace, the revised *Guidelines for the Maintenance and Promotion of Workers’ Health in Workplaces* was publicly announced for implementation from April 2021 [5]. Due to these national policies, private companies have come to attach more importance to efforts that promote health in the workplace.

In addition to increasing the risk of developing many mental and physical disorders, SB has been associated with declining job performance, poor work engagement (WE), and low presenteeism [6,7]. Thus, maintaining and improving workers’ health could improve the vitality of employees, invigorate the workplace, and improve productivity. In a study focusing on the relationship between the effects of physical activity (PA) on WE, which evaluated productivity improvement, it was reported that WE was higher in the group that performed physical exercise, such as calisthenics, at least once a week in the workplace [8]. Another report noted that having short sitting times during the workday could produce a better WE index of vitality for both sexes studied and enthusiasm in female employees [9]. Further, it has been indicated that employees with higher levels of health risk tend to suffer a greater loss of work productivity due to a decline in performance (presenteeism) caused by poor physical conditions [10]. Neck pain, shoulder stiffness, lower back pain, and mental health issues have also been noted as common reasons for decreased work performance. However, research on improving musculoskeletal symptoms due to oversitting lacks consistency in evidence due to the different intervention methods and periods that have been used and studied [11].

The general findings, from a health management perspective, indicate that increasing PA and reducing SB is important. Therefore, standing during work has begun to attract attention as a possible countermeasure against oversitting during the workday. The findings from an intervention study aimed at reducing SB during working hours in England via randomized controlled trials suggest that introducing sit–stand desks in offices reduces sitting time and improves general health effects [12]. However, in Japan, research related to SB and improvement in general workplace environments has only been conducted intermittently. Of the studies available, one demonstrated that office remodeling, including the introduction of sit–stand desks, could increase PA and lead to weight loss and improved work efficiency [13]. Conversely, it has been noted that office remodeling places a large financial burden on companies. The verification of the effect of introducing cheaper and “easier-to-use” sit–stand desks requires the accumulation and establishment of evidence comprising work-related indicators as outcomes when promoting reducing SB and conducting research in the field of occupational health. 

Therefore, this study assesses and verifies the effect of introducing a workplace sit–stand desk on Japanese workers’ general PA-, health-, and work-related outcomes. 

## 2. Materials and Methods

### 2.1. Study Participants and Design

This study used a randomized controlled trial and was conducted as part of a health promotion initiative by the university to which the first author belongs, in cooperation with Company R, which deals in Japanese electronic equipment. Participant recruitment for this study was conducted by selecting desk workers from among those who participated in the company’s health projects and belonged to either the technical department or the general affairs department. Participants with a history of serious complications (e.g., cardiovascular disease or cerebrovascular disease) were excluded from the recruitment process. 

**Figure 1:**
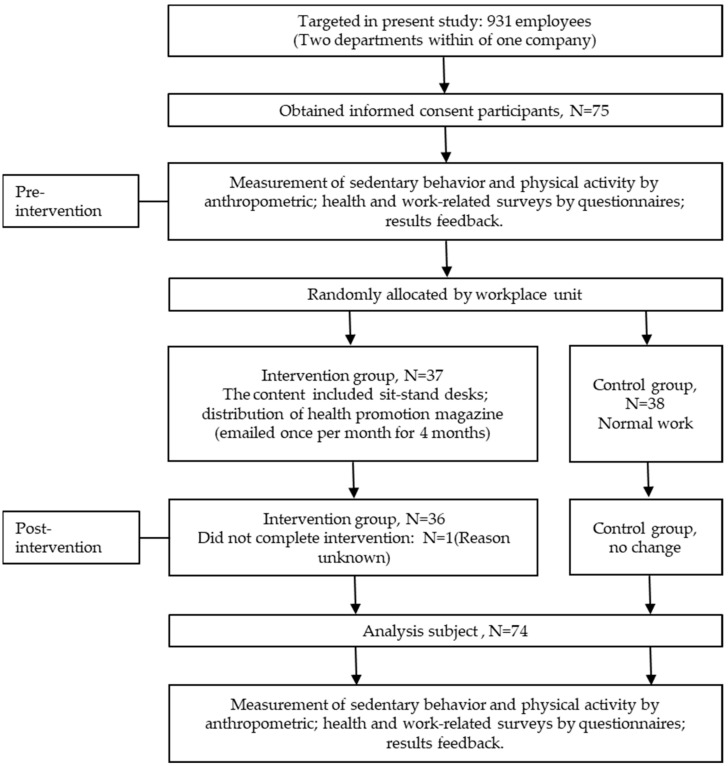
Flow chart of participants and protocol of intervention.

Of the 931 employees working at Company R, 75 desk workers agreed to participate in the three-month research study intervention. The participants from the technical department (*n* = 37) and general affairs department (*n* = 38) were randomly assigned to the intervention or control groups. Randomization occurred at the company level after baseline data were collected. One participant from the intervention group who exited the study was excluded. The final analysis included 36 and 38 participants in the intervention and control groups, respectively (Figure 1). 

Before the study was conducted, the working hours of both groups were confirmed to be around 7 hours and 30 min per day. There were no differences in PA and anthropometrics between the groups at baseline (Table 1). Before commencing data collection, the subjects were briefed on the purpose of the measurement, benefits, disadvantages, risks, and disclosure of data. Written informed consent was obtained from the participants. The implementation of this study was approved in advance by the Sendai University’s Research Ethics Review Board (approval number: 2019-12).

### 2.2. Intervention

Each participant in the intervention group received a personal sit–stand desk that allowed them to switch between sitting and standing positions and set their desk height freely during work hours. These participants were then asked to conduct their work as usual. Before and after the intervention, four health promotion magazines were provided once a month to the intervention group via a group email sent through a representative of each department. The content of these magazines included information about PA and health, including articles related to SB. 

### 2.3. Measures and Outcome Criteria

Characteristics of the participants: Demographic information collected by the questionnaires included items on gender, age, educational background, and marital status. For age, participants were asked to select between two age groups (20–39 or 40–59 years old); for educational level, participants could choose either four years or more of university-level education, or two years or fewer of university-level education; and for marital status, the participants were asked to indicate whether or not they were married.

Health-related outcomes: Measurements were taken using InBody (InBody 470, Tokyo, Japan). The evaluation items used were: (1) body mass index (BMI) and (2) body fat percentage. A questionnaire was conducted regarding the participants’ subjective health and their neck, shoulder, and lower back pain, with reference to previous studies related to the health assessment of desk workers [14,15].

PA-related outcomes: A tri-axial accelerometer (Active Style Pro HJA-750C, Omron Health Care Co., Ltd., Kyoto, Japan) was used to measure the participants’ SB and PA. The participants were asked to wear the accelerometer on their waist for a 10-day workday period, starting at the beginning of each workday and ending when they left work for the day. As all the participants were instructed to only wear the accelerometer during working hours, the wear time was regarded as working hours.

The chosen device stores resultant acceleration information with a measurement range of ±6G and a resolution of 3mG. It was possible to measure light physical activities, such as SBs, with a high degree of accuracy [16,17]. If the accelerometer remained at zero for 60 min or more, it was considered that the participant was not wearing it. PA volume was assessed using the tri-axial accelerometer in relation to light daily life activity (1.6~2.9 Metabolic Equivalents (METs)), sitting time (1.5 METs or lower), and moderate or greater PA (3 METs or higher) every 10 s. The data for SB and PA per day were extracted by assuming that the subjects had worn the accelerometers for at least 6.5 h per day over four working days [18]. 

Work-related outcomes: Work engagement was assessed using the shortened Japanese version of the Utrecht Work Engagement Scale (UWES-9) [19,20]. The scale consists of three subscales with nine items that assess positive mental state for work, including measures of vigor, dedication, and absorption. This scale has high internal reliability (α = 0.91–0.92) and good test–retest reliability (ICC = 0.66) [20]. It is recommended that work engagement is treated as a unitary construct due to the high correlations (r = 0.75–0.98) among the three components [19]. Work performance was assessed on a scale of 1 (worst ever) to 10 (best possible) with the following questions: “On a scale from 0 to 10, where 0 is the worst performance a person could have at your job, and 10 is the performance of the best worker, how would you rate the performance of most workers in a job similar to yours?”, “How would you rate your job performance in the past year or two?”, and “How would you rate your overall job performance on the days that you worked in the past four weeks (twenty-eight days)?”. The mean and standard deviation (SD) of item scores for each subscale were calculated.

### 2.4. Statistical Analyses

Means and standard deviations were calculated for the collected data. For each group, comparisons of individual attributes between the groups were conducted using a χ^2^ test for categorical variables. A paired *t*-test was used to compare pre- and post-intervention PA, and health-related and work-related outcomes. A group–time interaction assessed with ANOVA was also used to examine pre- and post-intervention changes in PA, along with health-related and work-related outcomes in both groups. Cohen’s d was calculated to measure the effect size values, and their corresponding 95% confidence interval (95% CI) was calculated. SPSS v.28 was used for statistical processing, with the significance level set at *p* < 0.05.

## 3. Results

The participants’ characteristics are presented in Table 1. Most participants were men, over 40 years old, with four years or more of university education and married. There were no significant differences between the intervention and control groups with respect to gender, age, educational background, and marital status.

Table 2 illustrates the PA-related, health-related, and work-related outcomes before and after the intervention for both the intervention and control groups. After the three-month intervention, the intervention group data indicate that their sitting time at work (*p* = 0.002), proportion of sitting time at work (*p* = 0.005), and their neck and shoulder pain scores (*p* =0.001) had all significantly decreased. Their scores of subjective health (*p* = 0.002), vitality in work-related engagement (*p* < 0.001), and self-rated work performance over a four-week period (*p* = 0.017) significantly improved. Group–time interaction between intervention and control groups had a significant difference in terms of work sitting time percentages (*p* = 0.044), subjective health (*p* = 0.034), neck and shoulder pain (*p* = 0.018), vitality in work-related work engagement (*p* = 0.010), and self-assessments of work performance over a four-week period (*p* = 0.034).

## 4. Discussion

In recent years, sitting for long periods in the office has been recognized as potentially unhealthy, and using a sit–stand desk to improve sitting behavior has attracted more attention. This study examined the impact of introducing sit–stand desks in the workplace on SB, health outcomes, and the productivity of desk workers required to sit for long periods. At the baseline, sitting time during work hours was approximately 60% in both the intervention and control groups. After the three-month intervention, the control group demonstrated no change, while the intervention group demonstrated a reduction in total sitting time and in the proportion of sitting time during working time, which indicates an improvement since introducing the sit–stand desks. However, the intervention group’s gait, light physical activity, and MVPA demonstrated no change before or after the intervention, which suggests that a sit–stand desk was used during their reduced sitting activity time (i.e., these participants replaced sitting with standing). Previous studies that reviewed the effectiveness of introducing sit–stand desks in the workplace reported an average reduction in sitting time of 100 min per day [21,22]. However, the decrease in sitting time in this study appears to be less compared to that seen in previous studies. This discrepancy could be attributed to the frequent interruptions in SB that may contribute to an overall decrease in sitting time [23]. The current study did not specifically indicate continuous sitting time or interruptions in SB, which allowed the subjects to switch freely between sitting and standing. This means that it may have resulted in a difference between the intervention evaluation methods. Additionally, in other intervention studies, SB at the baseline accounted for approximately 70% of participants’ working hours [24,25]. However, in this study, the subjects’ baseline sitting behavior accounted for less than 60% of the total. This finding indicates that participants’ sitting time was already less than that of participants in previous studies, which may have limited the extent of the subsequent decrease. 

Regarding health outcomes, no intervention effect was found on obesity-related BMI or body fat percentages in an objective assessment. Recommendations for improving the general health of desk workers include reducing sitting hours by four hours per day through standing or participating in low-intensity activities [24]. As the energy consumed by standing is insufficient to improve the obesity index [26], interventions with higher-intensity PA, such as walking around the office or increasing activity volume, may be necessary in addition to introducing sit–stand desks. No change was found in the lower back pain index before or after the intervention. However, after the intervention, the participants’ subjective view of their health improved, and they reported that pain in their neck and shoulders was reduced compared to before the intervention. A similar workstation-based study [27] also reported that reducing sitting time at work (by approximately one hour) resulted in improved subjective health, including lower levels of neck and shoulder pain and improved mood. However, that study indicated no improvement in lower back pain, as assessed by a questionnaire. Consistent with previous findings, this study found that subjective health indicators other than lower back pain were improved by introducing a sit–stand desk. This study also distributed health-promotion magazines every month to participants in the intervention group. This made them more knowledgeable about the deleterious effects of sitting and more aware that standing deskwork was healthier than sitting deskwork. Although there have been studies in which a decrease in SB did not improve musculoskeletal symptoms [28], it is undeniable that improved subjective health may also lead to improved productivity. It is also possible that a reduction in SB through the introduction of a sit–stand desk could be evaluated as a positive health effect. In Scandinavia, for example, the use of standing desks is so widespread that 90% of all employees work on PCs at sit–stand desks. Conversely, in Japan, although interest is growing, standing work has not yet become widespread, and when sit–stand desks were first introduced “standing fatigue” and other concerns (e.g., being worried about what other people might think) were cited as possible inhibitory factors to the widespread adoption of these desks [29]. However, it is believed that after using a sit–stand desk for a certain period, employees’ bodies adapt and their psychological resistance is eliminated due to the change in their work environment (i.e., people come to a better understanding of why their old desks have been removed, which makes it easier for them to use a sit–stand desk). 

Oversitting is related to poor work productivity, poor work engagement, and decreased presenteeism and can have an overall negative impact on working conditions [30,31]. In this study, work-related indicators were examined in relation to sit–stand desk implementation. Scores for vitality in work engagement and participants’ self-assessment of their work performance over four weeks were higher in the intervention group than in the pre-intervention and control groups. A study on SB and work engagement based on age group suggested that the longer the sitting time was, the lower the value of work engagement would be, with a greater impact seen in middle-aged individuals than in younger people [32]. As more than 80% of the subjects in this study were middle-aged, reducing their sitting time by using sit–stand desks is believed to have provided them with more vitality in their work, which, in turn, made them more energetic overall. Conversely, although vigor in work engagement indicators positively impacted working conditions, the same could not be found in relation to the dedication and absorption in work engagement labor indicators. In a previous cross-sectional study, workers with shorter sitting times reported a higher job satisfaction and less fatigue. Additionally, higher job satisfaction was associated with higher productivity [33]. Thus, rather than a direct relationship between SB and productivity, oversitting could be seen as causing physical and mental health problems, thereby secondarily affecting productivity through its negative impact on employees’ health and job satisfaction. Future studies should focus on verifying these results and relationships. 

It should be noted that this study has several limitations. Although the study design was a randomized controlled trial, it was not personal, and the subjects were divided into intervention and control groups solely on a worksite basis. Additionally, regarding the content of the intervention, the participants in the intervention group were only provided with an installed sit–stand desk and did not receive training regarding its usage, such as SB duration or the number of interruptions. Therefore, it was not possible to identify how the duration and number of SB interruptions affected the participants’ health and productivity specifically. Depending on the intention and physical condition of the participants, it is probable that being able to use the sit–stand desks freely without restriction could reduce their physical and psychological stress and lead to the continued use of these desks [11,34]. Furthermore, regarding the provision of health promotion magazines for health education, the effect of this intervention was not evaluated by ascertaining whether participants had read the content or their levels of understanding. Finally, although the participants’ demographic variables were adjusted when examining the intervention effect, lifestyle and leisure activity variables were not included in this study, which may have affected its findings on obesity and productivity [35]. Carrying out adjustments for these confounders may lead to different results. Despite the noted limitations, this current study’s use of a randomized controlled trial approach enabled it to clarify the effectiveness of the use of sit–stand desks for reducing SB. These findings are useful in Japan, where such knowledge is currently limited. It also contributes to the maintenance and improvement of workers’ health and productivity in Japan.

## 5. Conclusions

This study examined the impact of introducing a sit–stand desk to reduce the sitting time for desk workers on employees’ health and work. After a three-month intervention, periods of work time spent sitting were reduced in the intervention group compared with the pre-intervention and control groups. Health outcomes improved in terms of participants’ subjective health and the pain they experienced in their necks and shoulders. Regarding work efficiency, subjects’ vitality in work engagement and self-assessment of their work performance improved. It has been suggested that the use of the sit–stand desk reduces sitting time and has a positive effect on health and productivity at work. Future research is required to clarify how best to use sit–stand desks feasibly and continuously. It will also be necessary to accumulate evidence on the effectiveness of frequent interruptions and determine the time allocated to SB at work to improve employees’ health and productivity.

## Figures and Tables

**Table 1:** Characteristics of the participants at baseline.

	Intervention Group		Control Group		
	*n* = 37	%	*n* = 38	%	*p*-Value
Sex					0.833
Men	31	83.8	26	68.4	
Women	6	16.2	12	31.6	
Age, years					0.548
20–39	7	18.9	10	27.0	
40–59	30	81.1	27	73.0	
Education level					0.572
Four years of university or more	27	73.0	20	54.1	
Two years of university or fewer	10	27.0	17	45.9	
Marital status					0.675
Married	29	78.4	32	84.2	
Unmarried	8	21.6	6	15.8	

**Table 2:** The physical activity-, health-, and work-related outcomes at pre-intervention and post-intervention in the intervention and control groups.

	Intervention Group				Control Group				Group × Time Interaction
	Pre-Intervention *n* = 37	Post-Intervention *n* = 36	Effect Size (95% CI)	*p*-Value	Pre-Intervention *n* = 38	Post-Intervention *n* = 38	Effect Size (95% CI)	*p*-Value	*p*-Value
Physical activity-related outcomes									
Sitting time, min/work time	259.9 ± 29.5	239.2 ± 42.2	−0.53 (−0.19, −0.88)	0.002	266.6 ± 47.1	262.4 ± 58.8	−0.08 (−0.24, 0.41)	0.615	0.152
Sitting time of ratio ^a^, work time	59.2 ± 13.2	52.8 ± 10.4	−0.49 (−0.15, −0.88)	0.005	61.0 ± 9.2	60.9 ± 13.1	−0.01 (−3.78, 3.98)	0.960	0.044
Wear time, min/work time	458.8 ± 89.8	461.9 ± 83.1	0.04 (−0.36, 0.28)	0.806	440.1 ± 58.5	436.8 ± 70.9	−0.47 (−0.27, 0.36)	0.775	0.978
Steps, steps/work time	4566 ± 1842	4660 ± 1674	0.04 (−0.36, 0.28)	0.780	3957 ± 1678	3590 ± 2091	0.20 (−1.85, 0.53)	0.228	0.281
LPA ^b^, min/work time	164.2 ± 71.3	179.3 ± 71.3	0.25 (−0.58, 0.08)	0.134	138.3 ± 44.1	140.5 ± 68.2	0.04 (−0.36, 0.28)	0.817	0.420
MVPA ^c^, min/work time	42.5 ± 16.8	44.1 ± 16.3	0.09 (−0.42, 0.23)	0.574	37.8 ± 14.1	35.2 ± 18.1	−0.15 (−0.18, 0.47)	0.375	0.269
Health-related outcomes									
BMI, kg/m^2^	25.2 ± 4.2	25.3 ± 4.1	0.31 (−0.62, 0.03)	0.088	23.3 ± 3.0	23.2 ± 2.9	−0.04 (−0.36, 0.28)	0.807	0.397
Body fat mass %	27.1 ± 6.4	27.9 ± 6.0	0.29 (−0.62, 0.04)	0.084	24.5 ± 7.0	26.1 ± 7.3	0.76 (0.28, 1.11)	0.002	0.840
Self-rated health	2.32 ± 1.00	3.05 ± 0.94	0.55 (0.89, 0.20)	0.002	2.78 ± 0.78	2.92 ± 0.22	0.18 (−0.49, 0.14)	0.281	0.034
Pain neck–shoulders	2.73 ± 0.92	2.14 ± 0.82	−0.58 (−0.23, −0.93)	0.001	2.51 ± 1.04	2.43 ± 1.12	−0.10 (−0.22, 0.42)	0.556	0.018
Pain back	2.14 ± 0.91	2.11 ± 0.93	−0.04 (−0.29, 0.36)	0.822	2.05 ± 0.94	2.19 ± 1.05	0.14 (−0.46, 0.18)	0.391	0.474
Work-related outcomes									
Vigor ^d^	2.51 ± 1.37	3.32 ± 1.26	0.69 (1.05, 0.33)	<0.001	2.73 ± 1.66	2.84 ± 1.55	0.09 (−0.41, 0.26)	0.562	0.010
Dedication ^e^	2.84 ± 1.21	3.16 ± 1.26	0.29 (−0.68, 0.03)	0.076	3.14 ± 1.68	3.19 ± 1.56	0.03 (−0.35, 0.28)	0.834	0.338
Absorption ^f^	3.24 ± 1.32	3.46 ± 1.28	0.19 (−0.51, 0.13)	0.254	3.46 ± 1.52	3.32 ± 1.56	−0.10 (−0.29, 0.42)	0.536	0.254
Most workers assessment ^g^	5.27 ± 1.73	5.57 ± 1.57	0.17 (−0.49, 0.16)	0.316	6.16 ± 1.59	5.84 ± 1.89	−0.16(−0.16, 0.48)	0.320	0.187
Self assessment, past 4 weeks ^h^	5.32 ± 1.84	6.14 ± 1.60	0.34 (0.07, 0.62)	0.017	5.76 ± 1.65	5.49 ± 1.69	−0.45 (−0.27, 0.36)	0.547	0.034

Note: ^a^ Ratio sitting time for wear time (total working time); ^b^ light physical activity; ^c^ moderate-to-vigorous physical activity; ^d^ work-engagement vigor; ^e^ work-engagement dedication; ^f^ work-engagement absorption; ^g^ work-performance assessment of most workers; ^h^ work performance (self-rated job performance) over the past four weeks.

## Data Availability

Data provided in this study are available upon request from the corresponding author.

## References

[1] Biswas A., Oh P.I., Faulkner G.E., Bajaj R.R., Silver M.A., Mitchell M.S., Alter D.A. (2015). Sedentary time and its association with risk for disease incidence, mortality, and hospitalization in adults: A systematic review and meta-analysis. Ann. Intern. Med..

[2] Seguin R., Buchner D.M., Liu J., Allison M., Manini T., Wang C.Y., Manson J.E., Messina C.R., Patel M.J., Moreland L. (2014). Sedentary behavior and mortality in older women: The Women’s Health Initiative. Am. J. Prev. Med..

[3] Dunstan D.W., Howard B., Healy G.N., Owen N. (2012). Too much sitting—A health hazard. Diabetes Res. Clin. Pract..

[4] Bauman A., Ainsworth B.E., Sallis J.F., Hagströmer M., Craig C.L., Bull F.C., Pratt M., Venugopal K., Chau J., Sjöström M. (2011). The descriptive epidemiology of sitting. A 20-country comparison using the International Physical Activity Questionnaire (IPAQ). Am. J. Prev. Med..

[5] Ministry of Health, Labour and Welfare of Japan (2021). Guidelines for the Maintenance and Promotion of Workers’ Health in the Workplace. https://www.mhlw.go.jp/hourei/doc/tsuchi/T210209K0020.

[6] Boles M., Pelletier B., Lynch W. (2004). The relationship between health risks and work productivity. J. Occup. Environ. Med..

[7] Walker T.J., Tullar J.M., Diamond P.M. (2017). The longitudinal relation between self-reported physical activity and presenteeism. Prev. Med..

[8] Jindo T., Kai Y., Kitano N., Tsunoda K., Nagamatsu T., Arao T. (2020). Relationship of workplace exercise with work engagement and psychological distress in employees: A cross-sectional study from the MYLS study. Prev. Med. Rep..

[9] Eguchi H., Inoue A., Kachi Y., Miyaki K., Tsutsumi A. (2020). Work engagement and work performance among Japanese workers: A 1-year prospective cohort study. J. Occup. Environ. Med..

[10] Espahbodi S., Bassett P., Cavill C., Freeth M., Hole J., Sengupta R. (2017). Fatigue contributes to work productivity impairment in patients with axial spondyloarthritis: A cross-sectional UK study. Clin. Exp. Rheumatol..

[11] Parry S.P., Coenen P., Shrestha N., O’Sullivan P.B., Maher C.G., Straker L.M. (2019). Workplace interventions for increasing standing or walking for decreasing musculoskeletal symptoms in sedentary workers. Cochrane Database Syst. Rev..

[12] Edwardson C.L., Biddle S.J.H., Clarke-Cornwel A., Stacy C., Davies M.J., Dunstan D.W., Eborall H., Granat M.H., Gray L.J., Healy G.N. (2018). A three arm cluster randomised controlled trial to test the effectiveness and cost-effectiveness of the SMART Work & Life intervention for reducing daily sitting time in office workers: Study protocol. BMC Public Health.

[13] Ma J., Ma D., Wang Q., Kim H. (2020). The effects of changes in workplace environment on sedentary behavior and work efficiency: A natural pre-post study. Exerc. Med..

[14] Gao Y., Nevala N., Cronin N.J., Finni T. (2016). Effects of environmental intervention on sedentary time, musculoskeletal comfort and work ability in office workers. Eur. J. Sport. Sci..

[15] Graves L.E.F., Murphy R.C., Shepherd S.O., Cabot J., Hopkins N.D. (2015). Evaluation of sit-stand workstations in an office setting: A randomised controlled trial. BMC Public Health.

[16] Oshima Y., Kawaguchi K., Tanaka S., Ohkawara K., Hikihara Y., Ishikawa-Takata K., Tabata I. (2010). Classifying household and locomotive activities using a triaxial accelerometer. Gait Posture.

[17] Ohkawara K., Oshima Y., Hikihara Y., Ishikawa-Takata K., Tabata I., Tanaka S. (2011). Real-time estimation of daily physical activity intensity by a triaxial accelerometer and a gravity-removal classification algorithm. Br. J. Nutr..

[18] Sato T.O., Hallman D.M., Kristiansen J., Skotte J.H., Holtermann A. (2018). Different autonomic responses to occupational and leisure time physical activities among blue-collar workers. Int. Arch. Occup. Environ. Health.

[19] Schaufeli W.B., Bakker A.B., Salanova M. (2006). The measurement of work engagement with a short questionnaire: A cross-national study. Edu. Psychol. Meas..

[20] Shimazu A., Schaufeli W.B., Kosugi S., Suzuki A., Nashiwa H., Kato A., Sakamoto M., Irimajiri H., Amano S., Hirohata K. (2008). Work engagement in Japan: Validation of the Japanese version of the Utrecht work engagement scale. Appl. Psychol..

[21] Shrestha N., Kukkonen-Harjula K.T., Verbeek J.H., Ijaz S., Hermans V., Pedisic Z. (2018). Workplace interventions for reducing sitting at work. Cochrane Database Syst. Rev..

[22] Alkhajah T.A., Reeves M.M., Eakin E.G., Winkler E.A., Owen N., Healy G.N. (2012). Sit-stand workstations: A pilot intervention to reduce office sitting time. Am. J. Prev. Med..

[23] Ryde G.C., Brown H.E., Gilson N.D., Brown W.J. (2014). Are we chained to our desks? Describing desk-based sitting using a novel measure of occupational sitting. J. Phys. Act. Health.

[24] Buckley J.P., Hedge A., Yates T., Copeland R.J., Loosemore M., Hamer M., Bradley G., Dunstan D.W. (2015). The sedentary office: An expert statement on the growing case for change towards better health and productivity. Br. J. Sports Med..

[25] Clemes S.A., O’connell S.E., Edwardson C.L. (2014). Office workers’ objectively measured sedentary behavior and physical activity during and outside working hours. J. Occup. Environ. Med..

[26] Verweij L.M., Coffeng J., van Mechelen W., Proper K.I. (2011). Meta-analyses of workplace physical activity and dietary behaviour interventions on weight outcomes. Obes. Rev..

[27] Pronk N.P., Katz A.S., Lowry M., Payfer R.J. (2012). Reducing occupational sitting time and improving worker health: The Take-a-Stand Project, 2011. Prev. Chronic. Dis..

[28] Coenen P., Healy G.N., Winkler E.A., Dunstan D.W., Owen N., Moodie M., LaMontagne A.D., Eakin E.A., O’Sullivan P.B., Straker L.M. (2018). Associations of office workers’ objectively assessed occupational sitting, standing and stepping time with musculoskeletal symptoms. Ergonomics.

[29] Danquah I.H., Kloster S., Holtermann A., Aadahl M., Bauman A., Ersbøll A.K., Tolstrup J.S. (2017). Take a Stand!—A multi-component intervention aimed at reducing sitting time among office workers-a cluster randomized trial. Int. J. Epidemiol..

[30] Grimani A., Aboagye E., Kwak L. (2019). The effectiveness of workplace nutrition and physical activity interventions in improving productivity, work performance and workability: A systematic review. BMC Public Health.

[31] Ma J., Ma D., Kim J., Wang Q., Kim H. (2021). Effects of Substituting Types of Physical Activity on Body Fat Mass and Work Efficiency among Workers. Int. J. Environ. Res. Public Health.

[32] Ishii K., Shibata A., Oka K. (2018). Work engagement, productivity, and self-reported work-related sedentary behavior among Japanese adults: A cross-sectional study. J. Occup. Environ. Med..

[33] Arnold A.E., Coffeng J.K., Boot C.R., van der Beek A.J., van Tulder M.W., Nieboer D., van Dongen J.M. (2016). The relationship between job satisfaction and productivity-related costs: A longitudinal analysis. J. Occup. Environ. Med..

[34] De Cocker K., Veldeman C., De Bacquer D., Braeckman L., Owen N., Cardon G., De Bourdeaudhuij I. (2015). Acceptability and feasibility of potential intervention strategies for influencing sedentary time at work: Focus group interviews in executives and employees. Int. J. Behav. Nutr. Phys. Act..

[35] Smith L., McCourt O., Sawyer A., Ucci M., Marmot A., Wardle J., Fisher A. (2016). A review of occupational physical activity and sedentary behaviour correlates. Occup. Med..

